# *Hemiboeaalbiflora*, a new species of Gesneriaceae from Guizhou, China

**DOI:** 10.3897/phytokeys.122.33783

**Published:** 2019-05-28

**Authors:** Zhaowen Wu, Zhiyou Guo, Chaoyi Deng, Zhenyu Li, Xiaoguo Xiang

**Affiliations:** 1 Jiangxi Province Key Laboratory of Watershed Ecosystem Change and Biodiversity, Institute of Life Science and School of Life Sciences, Nanchang University, 330031 Nanchang, Jiangxi, China Institute of Botany, Chinese Academy of Sciences Beijing China; 2 State Key Laboratory of Systematic and Evolutionary Botany, Institute of Botany, Chinese Academy of Sciences, 100093 Beijing, China Nanchang University Nanchang China; 3 College of Biological Sciences and Agriculture, Qiannan Normal College for Nationalities, 558000 Duyun, Guizhou, China Qiannan Normal College for Nationalities Duyun China; 4 Karst Area Development Institute of Qianxinan, 562400 Xingyi, Guizhou, China Karst Area Development Institute of Qianxinan Xingyi China

**Keywords:** *
Hemiboea
*, Gesneriaceae, limestone flora, new species

## Abstract

*Hemiboeaalbiflora* X.G.Xiang, Z.Y.Guo & Z.W.Wu, **sp. nov.**, a new species of Gesneriaceae from Guizhou, China, is described and illustrated. This species was previously listed informally as a variety of *H.gamosepala*, but it differs significantly from *H.gamosepala* by its 5-parted calyx from the base, longer peduncle, white corolla and longer pistil. Based on recent extensive observations, this new species is similar to H.cavalerieivar.paucinervis and *H.subcapitata* but differs from them by its longer petiole, larger involucre, white corolla and longer staminal filaments. The conservation status of this species is considered to be “Vulnerable” (VU) according to the IUCN Red List Categories and Criteria.

## Introduction

*Hemiboea* C.B. Clarke is a medium-sized genus of Gesneriaceae distributed in central to southern China, northern Vietnam and Southern Japan (Li and Wang 2004). Recently, nine new species and one new variety were found in Guangxi, Guizhou and Yunnan province of China (Li and Liu 2004; Wei 2010; Xu et al. 2010, 2012; Huang et al. 2011; Wen et al. 2011, 2013; Pan et al. 2012; Zhou et al. 2013; Zhang et al. 2014; Li et al. 2018; Chen et al. 2018). Meanwhile, Weber et al. (2011) transferred two species of the Chinese endemic genus *Metabriggsia* W. T. Wang (1983) to *Hemiboea*, based on molecular and morphological evidence. In addition, Huang et al. (2017) treated *H.subcapitata var. pterocaulis* Z.Y. Li as a distinct species *H.pterocaulis*, based on molecular and morphological evidence. In total, the genus *Hemiboea* comprises at least 36 species and 5 varieties.

**Figure 1.**
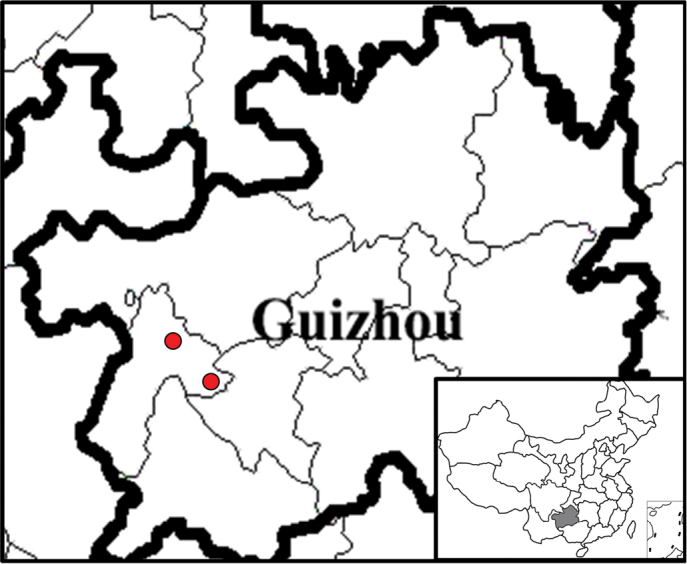
Distribution of *Hemiboeaalbiflora* in China.

During our expedition to Xingyi City, Guizhou Province, China in 2017, we collected two populations of Hemiboeagamosepalavar.albiflora C. Y. Deng & M. T. An, nom. nud. invalidly published in Deng and An (2006) (Fig. 1). However, we found that this variety differs significantly from *Hemiboeagamosepala* Z. Y. Li, especially by the calyx of the variety which is 5-parted from the base. After consulting *Hemiboea* specimens deposited in PE, KUN, IBK and QNUN and relevant literature (Li 1987; Wang 1983; Li and Wang 2004; Wei 2010), we concluded that it is a distinct species and hence we describe it as *Hemiboeaalbiflora*.

## Material and methods

Morphological observations and measurements of the new species were carried out, based on living plants and dry specimens (PE, QNUN and XIN). The photographs were taken in the field. All morphological characters were studied under dissecting microscopes and are described using the terminology presented by Wang et al. (1998).

## Taxonomic treatment

### Hemiboea albiflora

Taxon classificationPlantaeLamialesGesneriaceae

X.G.Xiang, Z.Y.Guo & Z.W.Wu sp. nov.

urn:lsid:ipni.org:names:60478837-2

Figs 2 , 3

#### Type.

China. Guizhou: Xingyi City, Maling River Valley, 26°8.47'N, 104°57.27'E, altitude 967 m, on rock faces near the river, 12 October 2017, *X.G. Xiang, Z.W. Wu & Z.Y. Guo 2017061* (holotype: PE!; isotypes: PE!, QNUN!).

#### Diagnosis.

*H.albiflora* differs significantly from *H.gamosepala* by its 5-parted calyx from the base (vs. 5-lobed from middle), longer peduncle, 3–6 cm (vs. 0.2–0.4 cm), white corolla (vs. pink corolla) and longer pistil, 2–2.5 cm (vs. ~ 1.5 cm). After extensive observations, *Hemiboeaalbiflora* is close to H.cavalerieivar.paucinervis W. T. Wang et Z. Y. Li and *H.subcapitata* C.B. Clarke, but differs from them through its longer petiole, 3–6 cm; larger involucre, 2–3 cm in diameter; white corolla, glabrous outside; and longer staminal filaments, 1.8–2 cm long. The detailed morphological comparisons are listed in Table 1.

**Table 1.** Morphological comparisons between *H.albiflora* and the similar species *H.gamosepala*, *H.cavalerieivar.paucinervis* and *H.subcapitata*.

	* H. albiflora *	* H. gamosepala *	H. cavaleriei var. paucinervis	* H. subcapitata *
**Leaf**				
Petiole	3–6 cm	0.6–6 cm	0.5–6.5 cm	0.5–5.5 cm
adaxial surface	green, sparsely pubescent	deep green, glabrous	green, glabrous	deep green, glabrous or sparsely pubescent
abaxial surface	pale green, glabrous	pale green or pale purple, glabrous	pale green or purple, glabrous	pale green, glabrous or sparsely pubescent
Veins on each side of midrib	5–9	4–10	4–8(-9)	5–6
**Flower**				
Peduncle	2–3 cm long	0.2–0.4 cm long	0.5–6.5 cm long	2–4(-13) cm long
Involucre	2–3 cm in diameter	1.8–2.3 cm in diameter	1–2.5 cm in diameter	1.5–2.2 cm in diameter
Corolla	4–5.5 cm long, outside white, glabrous	3.8–4 cm long; outside pink, sparsely glandular-puberulent	3.0–4.8 cm long, outside white, pale yellow or pink, sparsely glandular-puberulent	3.5–4.2 cm long, outside white, sparsely glandular-puberulent
Tube	3.5–4.5 cm long	3–3.1 cm long	2.3–3.3 cm long	2.8–3.5 cm long
Filaments	1.8–2 cm long	1.2–1.5 cm long	1.0–1.3 cm long	0.8–1.3 cm long
Anther	ovate-elliptic, 2–3 mm long	subovate, ca. 3 mm long	elliptic, 3–3.2 mm long	elliptic, 3–4 mm long
Staminodes	2	2	2	3
Pistil	2–2.5 cm long	ca. 1.5 cm long	1.7–2.5cm long	3.2–3.5 cm long
Capsule	2–3 cm long	1.8–2.4 cm long	1.5–2.5 cm long	1.5–2.2 cm long

#### Description.

Perennial herbs. Stems ascending, subterete, 40–100 cm tall, 2–5 mm in diameter, simple, sparsely purple-spotted, glabrous, juicy when fresh, nodes 5–10, not swollen. Leaves opposite, herbaceous; petiole 3–6 cm long, about 2 mm in diameter, almost terete, adaxially valleculate, margin erect and rounded, glabrous, green; leave blade oblong-lanceolate or ovate-lanceolate, 7–15 cm long, 3–5.5 cm wide, apex acuminate, rarely acute, margin repand-crenate, base usually oblique, adaxial surface green, sparsely pubescent, abaxial surface pale green, glabrous; lateral veins 5–9 on each side of midrib. Cymes subterminal, sometimes axillary, 4–8-flowered; peduncle 2–3 cm long, 3–4 mm in diameter glabrous, sparsely purple-spotted; involucre subglobose or broad ovoid, 2–3 cm in diameter, yellow-green, glabrous, apex acute. Pedicel 3–5 mm long, 2–3 mm in diameter, glabrous. Calyx white, 5-parted from the base, lobes equal, ovate-lanceolate, 1.2–1.6 cm × 0.3–0.4 cm, glabrous. Corolla white, with mauve lines and spots inside, 4–5.5 cm long, glabrous. Corolla tube 3.5–4.5 cm long, 1–1.4 cm in diameter at the mouth, 4–5 mm in diameter at the base, purple-spotted at throat, densely glandular-pubescent inside adaxial gibbous side of the tube, inside with a ring of hairs adnate to 7–8 mm above the corolla tube base; limb 2-lipped; adaxial lip 0.8–1.0 cm long, 2-lobed at apex, lobes equal, nearly semi-orbicular, abaxial lip 1–1.2 mm long, 3-parted, lobes subequal, oblong. Stamens 2, anthers fused by adaxial surfaces, adnate to 0.8–1 cm above the corolla base; filaments linear, glabrous, geniculate from the middle, 1.8–2 cm long, about 1 mm wide; anthers ovate-elliptic, glabrous, ca. 2–3 mm long, 2 mm wide, coherent at apex. Staminodes 2, glabrous, adnate to 1.2–1.5 cm above the corolla base, thick, 1.2–1.4 cm long, about 1 mm wide, apex capitellate, separate. Pistil 2–2.5 cm long; ovary linear, 0.7–0.9 cm long, 1.5–2.2 mm in diameter, glabrous; style 1.3–1.6 mm long, glabrous; stigma capitate, about 2 mm in diameter. Capsule linear-lanceolate, 2–3 cm long, 2–4 mm in diameter, glabrous, slightly curved.

**Figure 2.**
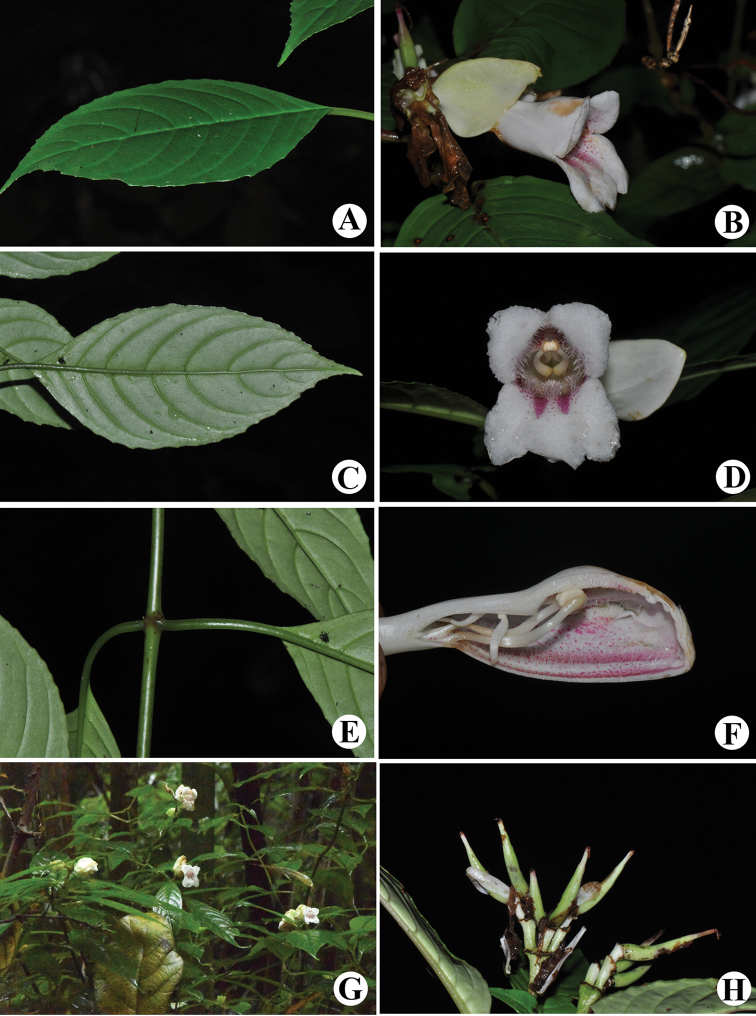
*Hemiboeaalbiflora*. **A** Adaxial leaf blade **B** flower side view **C** abaxial leaf blade **D** flower face view **E** stem and petioles **F** opened corolla showing stamens, staminodes and pistil **G** flowering habit **H** fruits. Photographs by Zhiyou Guo.

**Figure 3.**
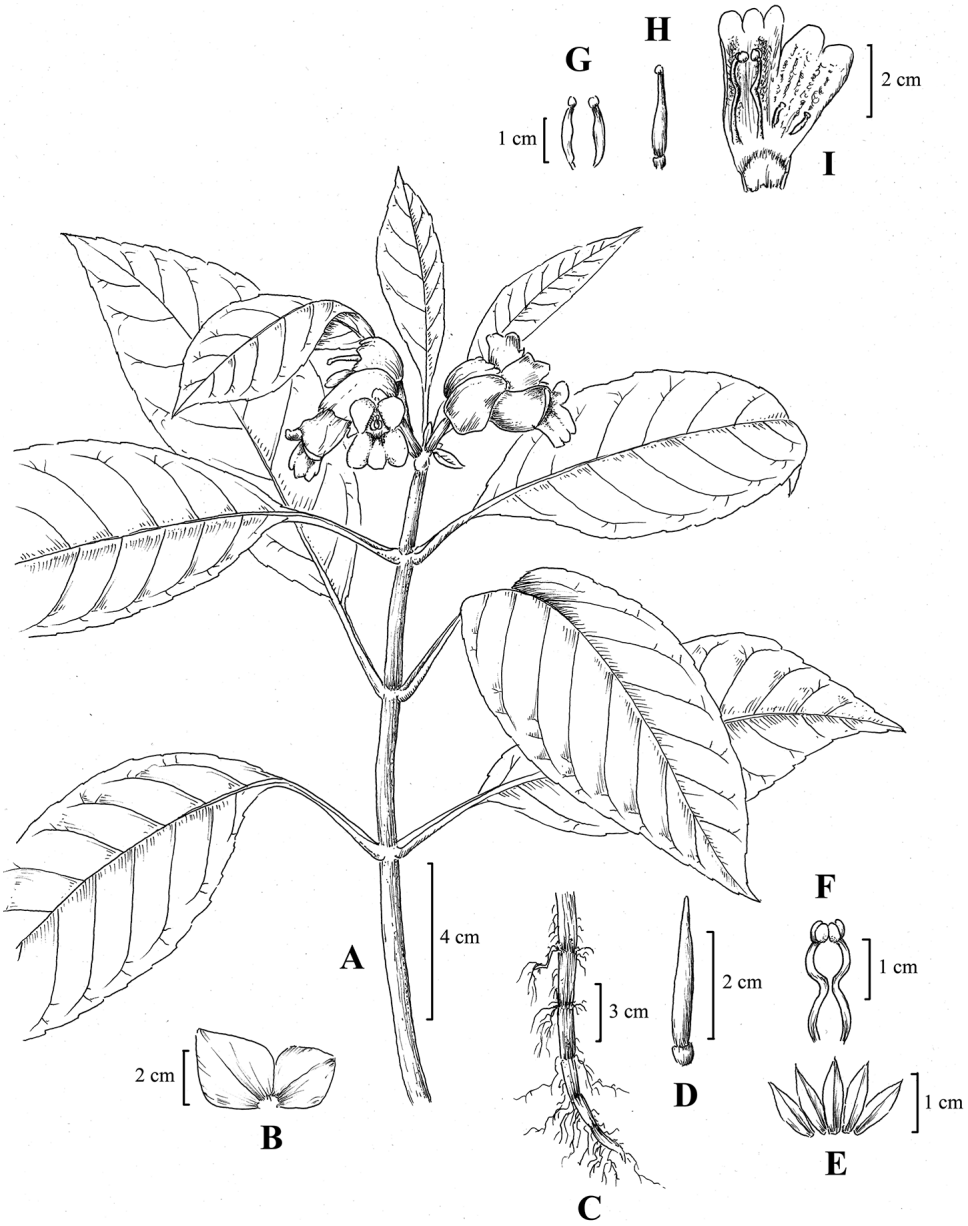
*Hemiboeaalbiflora*. **A** Flowering habit **B** involucre **C** root **D** capsule **E** calyx segments **F** stamens **G** staminodes **H** pistil **I** flower inside view. Drawn by Zhaowen Wu.

#### Distribution and habitat.

*Hemiboeaalbiflora* is known from Maling River Valley and Pogang Nature Reserve, Xingyi City, Guizhou, China, growing on rock faces near Maling River and near streams in Pogang Nature Reserve, at an elevation of ca. 720–970 m.

#### Phenology.

Flowering occurs in August to October and fruiting in October to November.

#### Etymology.

The specific epithet refers to the corolla colour of this new species.

#### Additional collection.

CHINA. Guizhou: Xingyi City, Maling River Valley, ca. 950 m alt., 12 October 2018, *X.G. Xiang, Z.W. Wu & Z.Y. Guo 2017060* (PE), *X.G. Xiang, Z.W. Wu & Z.Y. Guo 2017062* (PE); Xingyi City, Maling River Valley, ca. 720 m alt., 12 October 2018, *X.G. Xiang, Z.W. Wu & Z.Y. Guo 2017057* (PE); Xingyi City, Maling River Valley, *C.Y. Deng 3071* (XIN); Xingyi City, Pogang Nature Reserve, ca. 1000 m alt.,11 October 2018, *X.G. Xiang, Z.W. Wu & Z.Y. Guo 2017054* (PE), *X.G. Xiang, Z.W. Wu & Z.Y. Guo 2017055* (PE), *X.G. Xiang, Z.W. Wu & Z.Y. Guo 2017056* (PE).

## Proposed IUCN conservation status

To date, *Hemiboeaalbiflora* has two known populations of more than 300 and less than 1000 mature individuals, according to field observations. Both populations are endemic in karst areas and grow on rock faces or under forests near streams. The population, which is distributed in scenic spots and habitats, is susceptible to human activities, e.g. road construction or deforestation. The species is considered to be “Vulnerable” (VU D1) according to the IUCN Red List Criteria (IUCN 2017), based on Criterion D1 and population size, estimated to be fewer than 1000 mature individuals.

## Supplementary Material

XML Treatment for Hemiboea albiflora

## References

[B1] Chen WH Zhang YM Li ZY Nguyen QH Nguyeen TH Shui YM (2018) *Hemiboeacrystallina*, a new species of Gesneriaceae from karst regions of China and Vietnam. Phytotaxa 336(1): 95–99. 10.11646/phytotaxa.336.1.8

[B2] Deng CY An MT (2006) A name list of the seed plant in Pogang Nature Reserve, Guizhou. In: Zhang HH, Long QD, Liao DP (Eds) Proceedings of comprehensive scientific investigation of Pogang Nature Reserve in Xingyi City. Guangxi Sciences and Technology Publishing House, 79 pp. [in Chinese]

[B3] Huang YS Xu WB Peng RC Liu Y (2011) A new variety of *Hemiboea* (Gesneriaceae) from limestone areas in Guangxi, China. Taiwania 56: 240–243.

[B4] Huang J Xiang XG Lu YB Pan B Zhang Q (2017) *Hemiboeapterocaulis* comb. & stat. nov. (Gesneriaceae), a new species segregated from *H.subcapitata* C. B. Clarke. Nordic Journal of Botany 36(1_2): njb–01468. 10.1111/njb.01468

[B5] IUCN (2017) Guidelines for using the IUCN Red List Categories and Criteria. Version 13. IUCN Standards and Petitions Subcommittee. http://www.iucnredlist.org/documents/RedListGuidelines.pdf

[B6] Li ZY (1987) A study of the genus *Hemiboea* (Gesneriaceae). Zhiwu Fenlei Xuebao 25: 81–92.

[B7] Li ZY Liu Y (2004) *Hemiboearubribracteata* Z. Y. Li & Yan Liu, a new species of *Hemiboea* (Gesneriaceae) from Guangxi, China. Zhiwu Fenlei Xuebao 42: 537–540. [in Chinese]

[B8] Li ZY Wang YZ (2004) Plants of Gesneriaceae in China. Henan Sciences & Technology Publishing House, Zhengzhou. [in Chinese]

[B9] Li SW Han MQ Li XJ Li ZY Xiang XG (2018) *Hemiboeasuiyangensis* (Gesneriaceae): A new species from Guizhou, China. PhytoKeys 99: 99–106. 10.3897/phytokeys.99.25265 PMC5990545 29881323

[B10] Pan B Wu WH Xu WB (2012) *Hemiboeapseudomagnibracteata* (Gesneriaceae), a new species from Guangxi, China. Taiwania 57: 188–192.

[B11] Wang WT (1983) Genus novum Gesneriacearume Guangxi. Guihaia 3: 1–6.

[B12] Wang WT Pan KY Li ZY Weitzman AL Skog LE (1998) Gesneriaceae. In: Wu ZY Raven PH (Eds) Flora of China, Vol.18. Science Press and Missouri Botanical Garden Press, Beijing and St. Louis, 294–301.

[B13] Weber A Wei YG Sontag S Möller M (2011) Inclusion of *Metabriggsia* into *Hemiboea* (Gesneriaceae). Phytotaxa 23(1): 37–48. 10.11646/phytotaxa.23.1.2

[B14] Wei YG (2010) Gesneriaceae of South China. Guangxi Sciences and Technology Publishing House, 174–217. [in Chinese]

[B15] Wen F Tang WX Wei YG (2011) *Hemiboeaangustifolia* (Gesneriaceae), a new species endemic to a tropical limestone area of Guangxi, China. Phytotaxa 30(1): 53–59. 10.11646/phytotaxa.30.1.4

[B16] Wen F Zhao B Liang GY Wei YG (2013) *Hemiboealutea* sp. nov. (Gesneriaceae) from Guangxi, China. Nordic Journal of Botany 31(6): 720–723. 10.1111/j.1756-1051.2013.01697.x

[B17] Xu WB Wu WH Nong DX Liu Y (2010) *Hemiboeapurpurea* sp. nov. (Gesneriaceae) from a limestone area in Guangxi, China. Nordic Journal of Botany 28(3): 313–315. 10.1111/j.1756-1051.2009.00722.x

[B18] Xu WB Huang YS Peng RC Zhuang XY (2012) *Hemiboeasinovietnamica* sp. nov. (Gesneriaceae) from a limestone area along the boundary of Sino-Vietnam. Nordic Journal of Botany 30(6): 691–695. 10.1111/j.1756-1051.2012.01340.x

[B19] Zhang LX Tan YH Li JW Wen B Yin JT Lan QY (2014) *Hemiboeamalipoensis*, a new species of Gesneriaceae from southeastern Yunnan, China. Phytotaxa 174(3): 165–172. 10.11646/phytotaxa.174.3.5

[B20] Zhou SB Hong X Wen F Xiao HW (2013) *Hemiboearoseoalba* S.B. Zhou, X. Hong & F. Wen (Gesneriaceae), a new species from Guangdong, China. Bangladesh Journal of Plant Taxonomy 20(2): 171–177. 10.3329/bjpt.v20i2.17391

